# Engagement of Academic Staff Amidst COVID-19: The Role of Perceived Organisational Support, Burnout Risk, and Lack of Reciprocity as Psychological Conditions

**DOI:** 10.3389/fpsyg.2022.874599

**Published:** 2022-05-06

**Authors:** Melissa Reynell van der Ross, Chantal Olckers, Pieter Schaap

**Affiliations:** Department of Human Resources, Faculty of Economic and Management Sciences, University of Pretoria, Pretoria, South Africa

**Keywords:** engagement, perceived organisational support, job demands, lack of reciprocity, burnout risk, psychological well-being, academic staff

## Abstract

The COVID-19 crisis has resulted in radical changes within the higher education system, requiring academia to rapidly transition from the traditional learning model to a distance or blended model of learning to ensure continuity of educational processes. These changes have placed additional demands on academic staff who already have a heavy workload. According to the job demands-resources model, these additional demands may have an impact on the burnout risk, engagement, and well-being of academic staff. In alignment with the premises of positive psychology the primary objective of this study was to explore the interplay of three psychological conditions (meaningfulness, safety, and availability) needed to stimulate engagement. To investigate this interplay, the researchers connected Kahn’s theory on engagement with current concepts that focus on the person-role relationship, such as those dealt with in the job demands-resources model, organisational support theory, and perceptions of reciprocity. Mediating effects between burnout risk, engagement, and psychological well-being, as well as the moderating effect of lack of reciprocity, were tested using structural equation modelling. The study used a purposive, non-probability sampling method and a cross-sectional survey research design. Participants were 160 academic staff members employed at a university in South Africa. The findings of this study revealed that the three psychological conditions (meaningfulness, safety, and availability), which were operationalised as lack of reciprocity, perceived organisational support, and burnout risk, were significantly related to emotional engagement. Perceived organisational support (job resources), which met the criteria for psychological safety and some components of meaningfulness, displayed the strongest association with engagement. Policymakers within higher education institutions should be sensitive to the issues this study focused on, especially as regards the need to provide organisational support in times of crisis, such as the COVID-19 pandemic.

## Introduction

Higher education (HE) institutions play a key role in facilitating economic development and growth, and meeting the social needs of the 21st century (Boggs, 2003; Pouris and Inglesi-Lotz, 2014). The COVID-19 pandemic has, however, posed numerous challenges to employees (Liu et al., 2021) and organisations in all sectors. The pandemic has brought about changes requiring academia to rapidly transition from the traditional learning model to a distance or blended learning model to ensure continuity of educational processes (Ali, 2020; Armoed, 2021). In addition to coping with an already heavy workload, which include having to produce an increasing number of high-quality international publications (Barkhuizen et al., 2014), academic staff have had to offer extra support to students. This, due to abrupt changes in the academic calendar and the concomitant lack of physical interaction caused by the complete or partial change over from traditional face-to-face teaching to online or blended teaching (Chiu, 2021). Barkhuizen et al. (2014) asserted that all the demands made on academic staff may lead to their burnout and low levels of commitment.

Scholars have stated that even during times of change and uncertainty, engaging the workforce remains one of the key strategic imperatives to ensure success (Anthony-McMann et al., 2017) as it significantly affects essential business outcomes such as productivity, customer satisfaction, discretionary effort, commitment, and well-being (Shuck, 2011; Shuck and Reio, 2011, 2014). In a study that Chanana and Sangeeta (2020) conducted during the pandemic, they maintained that engagement is now, more than ever, a key factor in the success of organisations.

According to Kahn, the presence of three experiential or psychological conditions namely, meaningfulness, safety, and availability influence people to “employ” or express themselves (self-in-role) and personally engage. Kahn (1990) described personal engagement as an employee who harness themselves to their work role and express their “preferred self” physically, cognitively, and emotionally when they perform their work. He likened personal engagement to “self-employment” and described it as inspiring aspects that can be termed flow, intrinsic motivation, involvement, and mindfulness. Bailey et al. (2017) investigated the meaning, antecedents, and outcomes of engagement and found that most studies on the antecedents of engagement explored the experience of job-design-related factors, which included job demands or job resources. Accordingly, Mercali and Costa (2019) indicated that the job demands-resources (JD-R) model (Demerouti et al., 2001; Bakker and Demerouti, 2017) offers one of the most solid empirical foundations to clarify the psychological mechanisms that underlie engagement in a work context.

Drawing on Kahn’s (1990) theory and using the JD-R model as a framework, the present study explored the conditions that stimulated the positive psychological construct of engagement (Kotera and Ting, 2019) and investigated its role in contributing to optimal functioning (Gable and Haidt, 2005) in HE institutions.

## Literature Review and Hypotheses Development

### The Relationship Between Job Demands, Burnout Risk (Psychological Availability), and Engagement

The JD-R model stipulates that job demands refer to negatively valued physical, social, psychological, or organisational aspects that require continuous effort and cost or consume energy (Schaufeli and Taris, 2014; Bakker and Demerouti, 2017). Within the HE context, academic staff often need to reconcile the demands their teaching tasks and research work place on them. In addition to being responsible for teaching, administrative work, and community service, they are expected to conduct high-quality research (Taris et al., 2001; Houston et al., 2006); Taris et al. (2001) found that the time demands and pressure of having to do research and teach have a significant positive relationship with strain, which drains the energy of academic staff. Accordingly, they posited that the combination of teaching and research is a key source of stress for academic staff. Other studies conducted within an academic context conceptualised job demands (e.g., research, teaching, and administrative work) as time pressure (e.g., Skaalvik and Skaalvik, 2011) or as workload (Boyd et al., 2011). The stress resulting from time pressure and workload has been exacerbated by the changes and challenges staff have experienced because of the global COVID-19 pandemic (Liu et al., 2021). A major challenge in the HE context has been the need to shift from the traditional learning model to the distance or blended learning model to ensure continuity of educational processes (Ali, 2020; Armoed, 2021). In alignment with research carried out by Taris et al. (2001) and others (e.g., Boyd et al., 2011; Skaalvik and Skaalvik, 2011) within the context of education, the present study adopted the description of job demands as relating to: (1) time pressure, (2) relationships with colleagues, and (3) pressures stemming from teaching vs research tasks.

The JD-R model (Demerouti et al., 2001) outlines two psychological processes that are responsible for job demands and resources operating as antecedents to engagement and burnout. These processes include the energetic process and the motivational process (Schaufeli and Bakker, 2004; Jackson et al., 2006). According to the energetic process, job demands wear out and drain the energy of people, resulting in burnout or high levels of exhaustion. The JD-R model’s proposed energetic process seems to map well onto the strain coping mode described in Hockey’s (1997) state regulation model of compensatory control. This model takes into account different effects on observed performance under circumstances of high demands (e.g., workload or stress) and offers a framework for the analysis of issues associated with strain, fatigue, and psychological health. In the strain coping mode, individuals make an increased effort to accommodate the high demands, in this way maintaining levels of performance but at the cost of expending energy, which manifests itself psychologically in the form of exhaustion and/or physically in the form of an increased excretion of cortisol (which leads to, for example, burnout risk). In the passive coping mode, individuals’ perception of excessive demands results in a downward adjustment of performance objectives, for example, by reducing their level of accuracy or paying less attention so as to avoid the cost of expending more energy (e.g., through mental activity), which they perceive to be high already. Hockey indicated that complete disengagement from task goals may result in extreme forms of passive control. This suggests that high demands may lead to disengagement, which in turn may negatively affect performance. Rich et al. (2010), Han et al. (2020) and Rattrie et al. (2020) were in agreement. The scholars respectively found statistically significant negative associations between demands (hindrance demands) and engagement. Thus, based on the descriptions of the energetic process, the strain, and passive coping modes, and empirical work highlighted, the researchers formulated the following hypotheses for the present study:

H1: *There is a statistically significant positive relationship between job demands and burnout risk.*

H2: *There is a statistically significant negative relationship between job demands and engagement.*

The JD-R model’s energetic process, in which job demands wear out and drain the energy of people, resulting in burnout or high levels of exhaustion seems to link well with Kahn’s (1990) notion of (psychological) availability as referred to earlier. In Kahn’s (1990) description, the availability of people is dependent upon how well they cope with the demands of life, be it work or non-work related. Thus, how available people are to engage despite the distractions experienced as members of a social system. These distractions that shape availability include: depletion of physical and emotional energy; outside or personal lives (e.g., personal or non-work matters that drain or take away from one’s psychological availability); and insecurity (e.g., concerns about the quality of one’s work, how it compares with the work of others, and one’s status in the role that distracted one or “occupied energies”) (Kahn, 1990: p. 715). Burnout has been regarded as a metaphor for a state of mental weariness (Schaufeli and Bakker, 2004) or physical and emotional exhaustion (Kristensen et al., 2005). The Copenhagen Burnout Inventory, developed by Kristensen et al. (2005), operationalises burnout as consisting of fatigue and exhaustion. The questionnaire consists of three sub-dimensions, work-related burnout, client-related burnout, and personal burnout (Kristensen et al., 2005; Creedy et al., 2017). Hodson (2021) highlighted the importance of considering how the constructs we use within the field of psychology fit in with that already understood in the field. Based on this premise, and the highlighted linkages between the description of availability, the energetic process of how burnout risk is shaped, and the conceptualisation of burnout as per the Copenhagen Burnout Inventory, the researchers operationalised burnout risk as psychological availability.

The researchers also formulated a third hypothesis based on further findings in existing literature. The JD-R model further postulates that high levels of exhaustion threaten the energy resources of an engaged individual, which can impact levels of engagement negatively (Jackson et al., 2006; Bakker and Demerouti, 2017). In support of this postulation, Russell et al. (2020) indicated a significant negative relationship between burnout and work engagement. As regards Kahn’s (1990) theory, (psychological) availability was identified as one of three conditions that shape whether a person will personally engage or not. Availability refers to the psychological or physical resources people have available to enable them to engage despite the distractions experienced as members of a social system. Based on these criteria, the researchers hypothesised as follows:

H3: *There is a statistically significant negative relationship between burnout risk and engagement.*

### The Relationship Between Job Resources (Psychological Meaningfulness and Safety), Burnout Risk (Availability), and Engagement

The JD-R model explains that resources operate as antecedents to engagement (Schaufeli and Bakker, 2004; Jackson et al., 2006; Bakker and Demerouti, 2017) by way of a motivational process: the resources employees have available motivate them to be committed, have positive attitudes toward work (Albrecht, 2012), and contribute to work engagement (Demerouti et al., 2001; Bakker and Demerouti, 2017). The JD-R model’s motivational process seems to link well with the concept of perceived organisational support (POS). The concept of POS derives from the organisational support theory (Eisenberger et al., 1986) and describes the degree to which employees perceive that their employer cares about their well-being and values their contribution (Eisenberger et al., 2001; Eder and Eisenberger, 2008; Kurtessis et al., 2017). POS encapsulates the general beliefs employees hold regarding the commitment of the organisation towards them as a result of perceived beneficial or harmful treatment by the organisation. These beliefs are informed by organisational aspects e.g., traditions, practices, policies, job enrichment, as well as social aspects, e.g., receiving sincere praise and approval (Eisenberger et al., 1986). Similar to the premise of how resources motivate employees to be committed (Albrecht, 2012) and contribute to work engagement (Demerouti et al., 2001; Bakker and Demerouti, 2017); the held beliefs regarding POS influence work effort or behaviour. POS further fosters positive affective commitment toward the organisation (Kurtessis et al., 2017); and contributes to work engagement (Rich et al., 2010; Zacher and Winter, 2011). Furthermore, with reference to the statement of Bakker and Demerouti (2017, p. 312) that resources are not only needed to effectively perform work but are also “important in their own right,” POS can be regarded as a valued resource that helps employees carry out their work (Kraimer and Wayne, 2004).

Apart from the condition of psychological availability that Kahn’s (1990) grounded theory identifies as shaping personal engagement, the theory identifies the psychological conditions of safety and meaningfulness as necessary to stimulate personal engagement. Safety is experienced as feeling that one can express oneself without fear of negative consequences to one’s career or self-image and is influenced by supportive interpersonal relationships, group dynamics, management style, and organisational norms. Safety was thus promoted in the following cases: (1) where interpersonal relationships were supportive and trusting; (2) where the unconscious roles individuals assumed and perceived as per the group dynamics promoted a feeling of safety in bringing “their selves into” role performance. Here, Kahn (1990) referred to perceptions regarding the distribution of power and authority among groups, and how this could suppress individuals’ voices and negatively impact safety; (3) where the management style or processes were supportive, consistent, predictable and created paths along which employees could safely travel; and (4) where the organisational norms, general expectations, cues or boundaries could govern employees to safely execute work (Kahn, 1990, p. 710).

Scholars have put forth that employees tend to personify organisations, viewing line managers as organisational agents and their actions toward them as reflecting the intentions or actions of the organisation (Eisenberger et al., 1986; Karagonlar et al., 2016). Therefore, POS can be regarded as consisting of aspects that reflect management/interpersonal relationships, group dynamics, and (organisational) norms/expectations. This assumption is based on the following grounds: (1) POS captures aspects related to supportive and trusting interpersonal relationships by tapping whether the organisation or rather organisational agents consider the interest of the employee in decision making, offer help when the employee is in need and care about their well-being; (2) by tapping whether organisational agents notice extra effort, consider employee feedback and goals, or whether organisational agents would undermine or exploit the employee, POS captures considerations regarding treatment by organisational members with authority or power, and the room this allows the employee to safely bring “their selves into” role performance; (3) by tapping whether organisational agents value employee contributions, tries to make the job more interesting and cares about the employees’ work satisfaction, POS captures considerations regarding supportive management processes and opportunities for career growth; (4) by tapping the general beliefs regarding whether policies and governing practices are perceived as favourable, POS provides information regarding general norms which can inform appropriate or proportionate ways of working.

Meaningfulness is experienced when people feel they are valued, worthwhile, and not taken for granted (Kahn, 1990; Olivier and Rothmann, 2007). Factors that influence meaningfulness are whether tasks are challenging, allow for learning, and provide a sense of competence, whether employees’ role is central to/needed by the institution, and whether work interactions with co-workers or clients are meaningful (Kahn, 1990). POS includes facets of meaningfulness that tap into perceptions of not being taken for granted, working on challenging tasks, and performing a role that is of importance to an organisation. Thus, the present study operationalised POS as a job resource that included aspects of psychological meaningfulness and safety.

The possibility of POS as an antecedent to engagement has been considered within the business sector and within the HE context (e.g., Guan et al., 2014; Mabasa and Ngirande, 2015). Among staff at a business college, support was found that a relationship existed between POS and engagement (Najeemdeen et al., 2018); Kurtessis et al’s. (2017) view that POS should lessen burnout was confirmed in a study among academics that indicated that POS negatively affected levels of burnout (Yew and Ramos, 2019). In addition, results from a meta-analysis indicated a statistically significant association between job resources and both burnout and engagement (Rattrie et al., 2020). Based on these findings, the following hypotheses were formulated:

H4: *There is a statistically significant positive relationship between POS and engagement.*

H5: *There is a statistically significant negative relationship between POS and burnout.*

### The Influence of Reciprocity (Psychological Meaningfulness) on Engagement

According to equity theory, reciprocity is pursued in interpersonal or organisational relationships and denotes the equality of exchange between two parties (Schaufeli et al., 1996). Similarly, Rothmann and Welsh (2013) contended that social exchange relationships affect employee engagement. A study by Van Horn et al. (1999) put forth that teachers’ views that there exists disagreement between what they have invested and the outcomes/return received (e.g., in terms of student progress, gratitude or enthusiasm) can result in disillusionment and energy depletion. This suggests that the perception of lack of reciprocity might increase states of weariness such as burnout risk, a view that was corroborated by Bakker et al. (2000) who found that general practitioners’ perception of a lack of reciprocity had a positive impact on emotional exhaustion. Another example of the negative impact of lack of reciprocity is Eisenberger et al.’s (2014) finding that in cases where supervisors held the general view that subordinates would not reciprocate favourable treatment (highly reciprocation-wary supervisors), it weakened the positive relationship between supervisor POS and high-quality relationships with the subordinate (leader–member exchange). By implication, apart from job demands and burnout risk, perceptions of lack of reciprocity from student groups may further deplete the energy of educators, or result in educators being unable to reciprocate with engagement for the high POS received. Therefore, in alignment with considerations by Lorah and Wong (2018) that where the relationship between the independent variable (IV) and the dependent variable (DV) may differ or depend on the level of a third variable, called the moderator, lack of reciprocity was considered as a possible moderator in the relationship between the predictors of engagement (IV’s) and engagement (DV).

Lack of reciprocity seems to tick further criteria for Kahn’s (1990) psychological meaningfulness domain in terms of work interactions with clients and the perception of being valued or appreciated by this group, which did not seem to be covered by POS. Based on the above deductions made and following Kahn’s (1990) theory, the present study was able to test the coaction of psychological availability (burnout risk), safety (POS), and meaningfulness (POS and lack of reciprocity), by considering lack of reciprocity as a moderator in terms of the JD-R model’s proposed relationships relating to engagement. Accordingly, the following hypotheses were formulated:

H6a: *Perceived lack of reciprocity moderates the negative relationship between job demands and engagement, such that the relationship becomes stronger as lack of reciprocity increases.*
H6b: *Perceived lack of reciprocity moderates the positive relationship between POS and engagement, such that the relationship becomes weaker as lack of reciprocity increases.*
H6c: *Perceived lack of reciprocity moderates the negative relationship between burnout risk and engagement, such that the relationship becomes stronger as lack of reciprocity increases.*

### The Relationship Between Burnout Risk, Engagement, and Psychological Well-Being

Robertson and Cooper (2010) stated that the fostering of a culture associated with high performance and organisational effectiveness required the consideration of critical aspects such as engagement and psychological well-being. Wright et al. (2007) described psychological well-being as the overall effective psychological functioning of a person.

Hockey (1997) posited that the adjustments individuals make to deal with adverse conditions (e.g., high job demands) must take into account the need to maintain an acceptable state of well-being, in addition to considering the achievement of performance goals. These considerations seem to be reasonable cautionary measures individuals should take because later studies have suggested that: (1) job demands are linked to challenges related to well-being because of burnout (Schaufeli and Bakker, 2004; Jackson et al., 2006); (2) dimensions of burnout have a significant negative impact on psychological well-being (Wright and Hobfoll, 2004); and (3) participants with lower cortisol output have higher levels of psychological well-being (Ryff, 2013). Based on these findings, the following hypothesis was formulated:

H7: *There is a statistically significant negative relationship between burnout risk and psychological well-being.*

Shuck and Reio (2014) found that people who displayed high engagement had significantly higher levels of psychological well-being. Two relatively recent studies corroborated these scholars’ finding. In the first place, Jena et al. (2018) found that meaningful engagement allowed employees to feel positive toward their organisation and work, leading to psychological well-being. In the second place, Rusu and Colomeischi (2020) found a positive association between teacher engagement and well-being. Based on these findings, the following hypothesis was formulated:

H8: *There is a statistically significant positive relationship between engagement and psychological well-being.*

Important in the context of the present study was the association found between psychological well-being and important outcomes such as better job performance and mental and physical health (Wright and Cropanzano, 2000; Robertson and Cooper, 2010). This association was confirmed in studies that showed strong links between well-being and performance (Daniels and Harris, 2000; Lee, 2019). Moreover, Wright (2014) asserted that psychological well-being can be regarded as a robust determinant of good performance. Students are the primary recipients of the learning experience, and although much contested, university management regard student feedback as important for evaluating client satisfaction with educational programmes and lecturer’s performance (Tasopoulou and Tsiotras, 2017). Furthermore, using a measure from a different source than the lecturer offered an avenue to help control for method bias (Podsakoff et al., 2012). Accordingly, we formulated the following hypothesis:

H9: *There is a statistically significant positive relationship between psychological well-being and student-reported levels of lecturer performance.*

### The Mediating Role of Engagement and Burnout Risk

Studies have indicated that engagement plays a mediating role between antecedents and outcomes of engagement (Saks, 2006; Christian et al., 2011; Saks and Gruman, 2014). For example, Garg and Singh (2020) found that engagement mediated the association between work withdrawal behaviours and subjective well-being. A further finding was that engagement mediated the relationship between negative emotions and well-being (Rusu and Colomeischi, 2020). Based on these findings, the following hypothesis was formulated:

H10: *Engagement mediates the relationship between burnout risk and psychological well-being.*

In a study among teachers, Skaalvik and Skaalvik (2018) found that teacher well-being (measured in terms of exhaustion, feelings of a diminished or depressed mood, and psychosomatic responses) mediated the relationship between job demands and engagement. Similarly, Russell et al. (2020) found that burnout risk mediated the relationship between job demands and work engagement among educators in the United States. Accordingly, the researcher of the present study formulated the following hypothesis:

H11: *Burnout risk mediates the relationship between job demands and engagement.*

**FIGURE 1:**
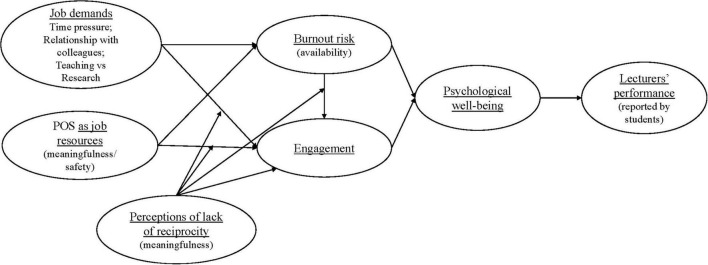
Conceptual framework (measurement model).

Figure 1 below provides the conceptual theoretical framework which is based on the above hypotheses.

## Materials and Methods

### Sample and Procedure

The study formed part of a bigger multilevel research project. Participating academic staff members thus needed to comply with the criterion of having lectured a second-semester undergraduate module during 2020. All in all, 295 academic staff members were invited during 2020, but, although 219 of them started the survey, only 174 valid responses were received. Students of participating lecturers were invited to report on the lecturers’ performance in lecturing the relevant modules. Out of the 174 valid responses received from lecturers, 161 could be matched with students’ reports. One statistically significant multivariate outlier was removed from the data sets prior to conducting the analyses using a conservative χ^2^ critical probability value of 0.001, resulting in a total sample of 160 lecturers. Males comprised 52% of the sample of academic staff, and females made up 48% of the sample. Most respondents (29%) fell within the age group category of 30 to 39, followed by 28% who fell within the age group of 50 to 64, and 26% who fell in the category of 40 to 49 years old. Respondents’ length of service in the various faculties ranged from periods of less than 5 years to over 31 years. A cross-sectional survey research design was employed, and a purposive, non-probability sampling strategy was used.

### Measures

The survey included the following measures:

#### Job Demands

In alignment with work that Taris et al. (2001), Skaalvik and Skaalvik (2011) did among academic staff, the following scales were used to measure job demands: the 3-item scale on time pressure; the 3-item scale measuring relations with colleagues; and the 4-item scale focusing on teaching vs research. In total, the scale consisted of ten items. Items for the scales of time pressure and relationship with colleagues were scored on a 6-point scale ranging from 1 = “completely disagree” to 6 = “completely agree.” The items relating to the teaching vs research scale were scored on a 6-point scale ranging from 1 = “never” to 6 = “always.” Cronbach’s alpha reported by Taris et al. (2001) was α = 0.84 (teaching vs research), and the reported reliability coefficients for scales reported by Skaalvik and Skaalvik (2011) were α = 0.86 (relationship with colleagues), and α = 0.81 (time pressure).

#### Job Resources

Job resources were measured using the 16-item short version of the “Survey of Perceived Organisational Support” (Eisenberger et al., 1997). Items were scored on a 7-point scale ranging from 1 = “strongly disagree” to 7 = “strongly agree.” The single-factor unidimensional measure demonstrated reliability coefficients of 0.90 (Eisenberger et al., 1997).

#### Engagement

The 18-item Job Engagement Scale (JES) (Rich et al., 2010) was used to measure engagement. The scale’s items measure three dimensions of engagement, namely, emotional, cognitive, and physical. Respondents could score the items on a 5-point rating scale ranging from 1 = “strongly disagree” to 5 = “strongly agree.” The JES has good internal consistency with a Cronbach’s alpha equal to 0.95 (Rich et al., 2010).

#### Burnout Risk

The 19-item Copenhagen Burnout Inventory (Kristensen et al., 2005) was used to measure burnout risk. The measure consists of three subscales, namely, personal, client, and work-related burnout. In respect of 12 items, the rating is on a 5-point Likert scale, ranging from 1 = “always” to 5 = “never/almost never,” and in respect of seven items the rating is on a scale of 1 = “to a very high degree” to 5 = “to a very low degree.” Cronbach’s alpha for the subscales was found to be as follows: α = 0.82 (client-related burnout), α = 0.85 (personal burnout), and α = 0.87 (work-related burnout) (Johnson and Naidoo, 2013).

#### Psychological Well-Being

The Schwartz Outcome Scale-10 (Blais et al., 1999) was used to measure psychological well-being. The scale, consisting of 10 items, has been used as a psychological well-being and psychological health measure in previous studies (e.g., Young et al., 2003; Haggerty et al., 2010). Items are rated on a 7-point scale ranging from 1 = “never” to 7 = “all of the time/nearly all of the time.” Cronbach’s alpha coefficients were reported between α = 0.88 (Haggerty et al., 2010) and α = 0.92 (Shuck and Reio, 2014).

#### Lecturer Performance

The lecturers’ performance was reported by students. The researcher adapted questions taken from the student evaluation form that the university used and that focused on lecturers’ characteristics and the conditions during lectures. The scale consisted of 22 items. A similar approach had been used in a study exploring student evaluations of lecturers at private universities (Sok-Foon et al., 2012).

#### Reciprocity of Student Groups

In alignment with work done by Schaufeli et al. (1996), Tayfur and Arslan (2013), lecturers’ perceptions of reciprocity were measured using three items adapted from the measures these scholars had used. The items were as follows: “I spend much time, effort and consideration on work for students in the specified module, but in general, students in the specified module give back little effort, appreciation, and interest”; “I invest more in the relationship with students in the specified module than what I receive back in return from them”; and “I know that my students will complain, no matter what I do.” Respondents rated the questions on the following 5-point scale: 1 = “strongly disagree” to 5 = “strongly agree.”

### Data Analysis

Mplus version 8.6 was used to conduct the statistical analyses. Latent models were estimated using structural equation modelling (SEM) and the maximum likelihood robust (MLR) estimator. The delta method for estimating robust standard errors with a sandwich estimator was used for non-normal data. As an extra precaution, all standard errors for interaction effects were cross-checked for consistency using the bias-corrected bootstrapping technique and the ML estimator (Muthén and Muthén, 1998–2017; Schaap and Olckers, 2020). Two stages were followed in the analysis of the data. First, confirmatory factor analysis (CFA) was used to confirm the factor structure validity and psychometric properties of each of the scales in order to ensure factor manifest scores with the least possible error variance. Factor scores that were used in the structural model were generated from the latent variable using the regression approach in Mplus. The use of optimally weighted regression scores entailed creating factor scores from the model for each construct separately and subsequently using these factor scores in the structural model (McNeish and Wolf, 2020). Second, an evaluation was done of the structural model depicting the theoretically supported hypothesised relationships between the constructs that formed the focus of this study. The two-stage approach and the use of optimally weighted regression factor scores in the structural model can be substantiated as follows: (1) The use of optimally weighted regression scores in a structural model alleviates potential convergence problems associated with the testing of complex latent structural models; (2) the quality of a measurement model affects the structural model and vice versa, even in the case of well-fitting measurement models (McNeish and Hancock, 2018); (3) McNeish and Wolf (2020) posited that possible differences in the relationship between items and the true latent score are ignored when using sum scoring or unit weighted scoring, resulting in less reliable scores. Optimally weighted regression scores thus more closely represent the true latent variable in the measurement model.

Following the guidelines suggested by Kenny et al. (2015), the present study considered model fit together with regression estimates, standard error, residuals, and underlying substantive theory. All the popular fit indices were considered and where degrees of freedom were low in models, the CFI and SRMR played a more decisive role in adjudicating model fit (Bentler, 2007; Kenny et al., 2015; McNeish and Hancock, 2018). Per the guidelines, model fit was appraised as: a CFI value above 0.90 but preferably above 0.95, a SRMR value preferably less than 0.08, a RMSEA value below 0.08, and a TLI value above 0.95 (Hu and Bentler, 1999; Olckers and Van Zyl, 2019).

The factor score determinacy is of special concern for unbiased univocal scoring of a measurement model (Gorsuch, 1983). Factor determinacies of 0.80 and above were regarded as demonstrating strong correlations among items with the latent factor and denoting good internal consistency (Gorsuch, 1983; Wang and Wang, 2020). Univariate normality and multivariate normality were tested for skewness and kurtosis and were appraised in alignment with the recommendation by Anderson and Gerbing (1988) that values equal to >-1 and <+1 in the case of both skewness and kurtosis be used as indicators of normality. Also, the Mardia multivariate normality test was used to evaluate the normality assumption.

In alignment with findings that the omega coefficient offers a more accurate approximation of the internal structure of a scale (Dunn et al., 2014; Crutzen and Peters, 2017), the present study used the CFA factor loadings to calculate McDonald’s omega coefficient. Values of 0.70 and 0.80 have been considered as the general rule of thumb when it comes to establishing acceptable or good reliability and have been commonly reported as the more popularly used Cronbach’s alpha estimates (Crutzen and Peters, 2017; Hoekstra et al., 2019). Seemingly, scholars have applied a similar rule in judging McDonald’s omega coefficient, putting forth that values of 0.80 can be regarded as demonstrating good internal reliability (Feisst et al., 2019; Dedeken et al., 2020). Confidence intervals (CIs) were set at a level of 95% and, as recommended in the case of interacting effects, the present study applied the guideline that where CIs did not include zero, the indirect effect was regarded as significant (Zhao et al., 2010).

## Results

### Descriptive Statistics

Table 1 shows the descriptive statistics, skewness/kurtosis, correlations, factor determinacy values and McDonald’s omega values of the latent variables. Most variables show univariate skewness and kurtoses slightly outside of the range −1, 0 to +1 (Anderson and Gerbing, 1988). The Mardia multivariate skewness and kurtosis are 9.73 and 69.62 respectively (see Table 1), these values indicate non-normality in the data (Gao et al., 2008), justifying the use of the MLR estimator for non-normal data. The correlation matrix indicated statistically significant relationships (*p* < 0.01) between all variables. McDonald’s omega coefficient values ranged between 0.81 and 0.97, demonstrating good reliability (Feisst et al., 2019; Hoekstra et al., 2019; Dedeken et al., 2020). Factor determinacy values were all above 0.90, demonstrating strong correlations among items with the latent factor (Wang and Wang, 2020) and supporting the use of factor scores in the structural model (Gorsuch, 1983). The results reported in Table 1 do not support the likelihood of adverse multi-collinearity as scale reliabilities are high (Omega ≥ 0.8) for the variables with high inter-correlations and sample size (*N* = 160) to the number of latent variables (6) exceeds a 6:1 ratio (Grewal et al., 2004). The variables in the correlation matrix show discriminate validity as all values below the diagonal are lower than the square root of the average variance extracted (AVE) which is presented on the diagonal (Fornell and Larcker, 1981).

**TABLE 1:** Descriptive statistics, correlations, skewness/kurtosis, and factor determinacy.

Variable		Skewness	Kurtosis	1	2	3	4	5	6	FD	ω
1	Burnout risk	−0.08	−0.83	0.85						0.98	0.96
2	Emotional engagement	−0.73	−0.07	−0.51	0.81					0.98	0.94
3	Psychological well-being	−1.02	0.43	−0.61	0.51	0.72				0.96	0.91
4	Job demands	−1.07	0.88	0.65	−0.36	−0.47	0.77			0.98	0.91
5	POS	−0.16	−0.03	−0.57	0.53	0.54	−0.43	0.80		0.98	0.94
6	Lack of reciprocity	−0.60	−0.63	0.33	−0.36	−0.27	0.32	−0.34	0.75	0.93	0.81
7	Lecturer performance	1.27	2.12	−0.05	−0.11	0.03	−0.05	−0.04	0.07	0.99	0.97

	**Mardia’s multivariate values**	**Estimate**	***p*-Value**								

	Mardia’s multivariate skewness	9.73	0.00								
	Mardia’s multivariate kurtosis	69.62	0.00								

*POS, Perceived organisational support; 160 participants made up the study sample; FD, Factor score determinacy; ω, McDonald’s omega. Factor scores are Z-values with a mean of 0. Underlined values on the diagonal represent the square root of the AVE (Fornell and Larcker, 1981). All correlations are statistically significant (p ≤ 0.05).*

### The Measurement Model

Table 2 provides an overview of the constructs measured and the fit indices per construct. No absolute cut-off value for factor loadings was used; however, the approach followed was “the higher the better,” with due consideration to item content and construct coverage. A special effort was made to retain as many as possible of the original items for each construct that proved to be psychometrically sound in the measurement models and that allowed for limited bias in the single and univocal score obtained for each measure used in this study (Cole and Preacher, 2014). As the scales used had been validated in previously published studies, the use of CFA rather than exploratory factor analysis was selected. However, where the data did not support model fit or item loadings, the models were re-specified in accordance with theoretical guidelines to ensure that robust psychometric measurements were obtained for the variables used in the study’s structural model. This is in alignment with Jackson et al. (2009) advice that *post hoc* modifications are supportable when these modifications are practically or theoretically justifiable. Recommendations in these cases include that any *post hoc* model re-specifications should be kept to a minimum as such re-specifications could erroneously lead to data-driven models, and that *post hoc* modifications be labelled, thus revealing which latent variables were allowed to correlate or which correlated residuals were freed. Measurement models that were not supported by the data were adapted in accordance with the following principles: (1) Jöreskog’s (1993) recommendation that by freeing a fixed or constrained parameter with the largest modification index (provided that this parameter can be interpreted substantively), the correct model can be readily obtained. (2) Byrne et al. (1989) advice that parameter specifications are justifiable where they represent measurement error due to method effects (e.g., item format of subscales). These method effects or measurement errors are attributed to question wording (e.g., items containing similar words, phrases or similar meaning), negative scoring, the effect of item adjacency, close proximity or blocked items from the same construct that follow each other in direct sequence (Podsakoff et al., 2012; Loiacono and Wilson, 2020). (3) Reise et al. (2013) recommendation to avoid biased path estimates in SEM models tested by not treating unidimensional data as multidimensional and to rather use only measurement models where the fit indices and variance explained support a sufficiently defined common or general factor that justifies univocal scoring.

**TABLE 2:** Fit statistics per measurement construct included.

Construct measured	Subscales	Items used	χ^2^	*df*	*p*	CFI	TLI	SRMR	RMSEA
(a) Job demands	Time pressure	2	35.73	8	0.00	0.94	0.90	0.05	0.14
	Relationship with colleagues	3 (*removed*)							
	Teaching vs research	4							
(b) Perceived organisational support (POS)	n/a	16	213.91	103	0.00	0.91	0.90	0.06	0.08
(c) Burnout risk	Personal burnout	5	147.04	43	0.00	0.93	0.91	0.04	0.12
	Work-related burnout	6							
	Client-related burnout	6 (*removed*)							
(d) Engagement	Physical engagement	*6* (*removed*)	22.17	9	0.01	0.98	0.96	0.02	0.09
	Emotional engagement	6							
	Cognitive engagement	*6* (*removed*)							
(e) Psychological well-being	n/a	8	36.38	20	0.01	0.97	0.96	0.04	0.07
(f) Lack of reciprocity	n/a	3	0.00	0	0.00	1.00	1.00	0.00	0.00
(g) Lecturer performance, measured by students using an adapted student evaluation form	n/a	22	1663.41	209	0.00	0.90	0.89	0.04	0.07

*χ^2^, chi-square statistic; df, degrees of freedom; p, p-Value; CFI, Comparative fit index; TLI, Tucker-Lewis Index; SRMR, Standardised root mean square residual; RMSEA, Root mean square error of approximation.*

The second-order measurement model for the job demands scale was non-identified and did not converge. It was found that the dimension of relationship with colleagues showed low correlations (*r* = 0.10; *r* = 0.03) with the dimensions of time pressure (*r* = 0.10) and teaching vs research (*r* = 0.03), suggesting that these constructs were unrelated. Upon reviewing the dimension of relationship with colleagues, it seemed that its items were formulated to contribute to job resources and not job demands (e.g., “Educators at this university help and support each other”). Consequently, a unidimensional model was tested that excluded this dimension. After excluding one item (which had a low factor loading of 0.28) and allowing the residuals of two items to correlate due to similar item content/method effects (Byrne et al., 1989; Podsakoff et al., 2012; Loiacono and Wilson, 2020) the model was supported by the data. It is noted that in accordance with the work of Kenny et al. (2015), the low degrees of freedom (8) did indeed result in an elevated RMSEA value (see Table 2).

POS was measured using a one-factor/unidimensional model consisting of 16 items. Two items, for which correlated residuals were allowed, scored negatively and were adjacent to each other, suggesting method effects (Byrne et al., 1989; Podsakoff et al., 2012; Loiacono and Wilson, 2020). The scale showed an acceptable model fit (see Table 2).

The construct of burnout risk consisted of three subscales (i.e., personal, work-related, and client-related) (see Table 2). Client-related burnout displayed low correlations with both personal (0.35) and work-related burnout (0.43), and it displayed low loadings (0.45) on the second-order model (showing low model fit), and was thus removed. The scales personal and work-related burnout correlated highly (*r* = 0.93) and were grouped as one unidimensional scale. For all practical purposes, these two constructs could not be considered separate in the case of the sample group as working from home was a general trend during the COVID-19 pandemic. Residuals for four items, were allowed to correlate because of one or more method effects/measurement error (Byrne et al., 1989; Podsakoff et al., 2012; Loiacono and Wilson, 2020). One item, the only negatively scored item that displayed low factor loadings (0.46), was removed, after which a good model fit was obtained (see Table 2).

Data from the study did not support a second-order factor measure for the JES that would produce a univocal and non-biased factor score; therefore, the researcher considered the core focus of Kahn’s (1990) theory, which is to simultaneously explain the emotional reactions of people to unconscious and conscious phenomena. Kahn entertained the possibility that a hierarchy of engagement or investment of the self in the work role exists, in that people may engage or invest themselves first physically, then cognitively, and lastly emotionally. Thus, the researcher explored this final level of the hierarchy (i.e., emotional engagement) and consequently excluded the cognitive and physical engagement dimensions from the measurement model. The retained emotional engagement subscale showed sufficient unidimensionality and model fit that would support univocal factor scoring and result in non-biased factor scores. The subscale consisted of six items and displayed good model.

The construct of psychological well-being, measured as a unidimensional scale, consisted of 10 items. Two items showed method effects (Byrne et al., 1989; Podsakoff et al., 2012; Loiacono and Wilson, 2020) and demonstrated high correlated residuals; thus, one item that showed redundancy was removed. Two items that demonstrated high correlated residuals which were attributed to method effects—were allowed to correlate (Byrne et al., 1989; Podsakoff et al., 2012; Loiacono and Wilson, 2020). The scale displayed acceptable model fit (see Table 2). The construct of lack of reciprocity consisted of three items; this three-item scale was a (just-) identified model (zero df) and displayed good model fit. An adapted version of the student evaluation form used by the university was employed to measure lecturer performance. The total score of the measure was used as a performance measure in practice (unit-weighted). The scale displayed acceptable model fit.

### The Structural Model and Hypotheses Testing

As indicated in Table 3, the hypothesised measurement model (Model 1) provided a poor fit to the data (CFI = 0.83; SRMR = 0.09; TLI = 0.73; RMSEA = 0.12). Furthermore, the lecturer performance scale showed an insignificant regression path (close to 0) on well-being. Kenny (2020) asserted that a good-fitting measurement model is required before researchers can endeavour to interpret a structural model, but warned that once model fit drives the research, scholars move away from theory testing, and the latter is the purpose of SEM (Hooper et al., 2008). This study formed part of a multi-level research project, thus alternative models were tested based on prior empirical work.

**TABLE 3:** Fit statistics of the path and alternative models.

Model	χ^2^	*df*	*p*-Value	CFI	TLI	SRMR	RMSEA
1	63.01	19	0.00	0.83	0.73	0.09	0.12
2	33.81	18	0.01	0.94	0.90	0.06	0.07
3	18.98	10	0.04	0.96	0.92	0.05	0.08

The hypothesised model (Model 1) was used as a template for Model 2; however, an indirect path was included, based on the finding of Russell et al. (2020) that burnout partially mediates the relationship between job resources and work engagement, and similar findings by Hakanen et al. (2006) that burnout mediates the relationship between POS (job resources) and emotional engagement. The modified Model 2 showed improved model fit (CFI = 0.94; SRMR = 0.06; TLI = 0.90; RMSEA = 0.07), but, considering the recommendation of Cohen (as cited by Hox et al., 2018) that a power of 0.80 with a corresponding β = 0.20 is a high power value, the power of 0.67 displayed by Model 2, (*N* = 160; *df* = 18, effect size = 0.10, α = 0.05) was insufficient. This finding was in alignment with the method of Satorra and Saris (1985) that recommends a power value of 0.80 to be desirable for SEM (Zhang and Yuan, 2018). To be more conservative, the researcher worked with an effect size of 0.10, which would provide for sufficient coverage of interaction effects that were inclined to be small but statistically significant. Model 2 served as a template for Model 3; however, the lecturer performance scale was excluded due to low power and the lecturer performance scale’s demonstration of an insignificant regression path (close to 0) on well-being. After removal of the lecturer performance scale, improved overall model fit (CFI = 0.96; SRMR = 0.05; TLI = 0.92; RMSEA = 0.08) and sufficient power of 0.79 (*N* = 160; *df* = 10, effect = 0.10, α = 0.05) was obtained. The researcher noted that statistical interaction effects lower than 0.10 were likely to not be recognised. The results obtained suggested that the empirical data were reproduced reasonably well in respect of the measurement models.

Model 3, which showed the best fit and power, formed the basis of the structural model. It should be noted that in the reporting of the results, significant implied “statistically significant.”

**FIGURE 2:**
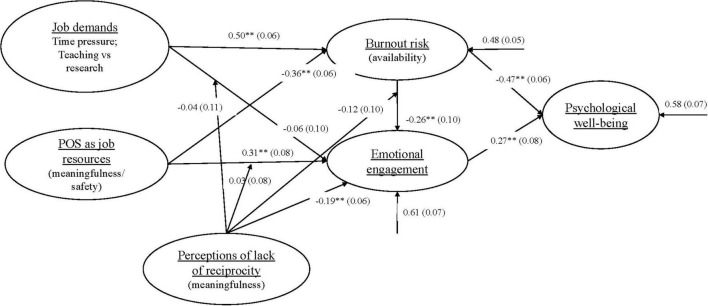
The path/structural model tested Note. **p < 0.01.

As displayed in Figure 2 (the portion of the model predicting burnout risk), the direct effect of job demands (β = 0.50, *p* < 0.01) was significantly positive (large effect), providing support for hypothesis 1. The effect of POS (β = −0.36, *p* < 0.01) was significantly negative (medium effect), providing support for hypothesis 5. Job demands and POS explained 52% of the variance in burnout risk (*R*^2^ = 0.52).

As displayed in Figure 2 (the portion of the model predicting emotional engagement), the direct effect of job demands (β = −0.06, *p* = 0.57) was not statistically significant; therefore, hypothesis 2 was not supported. Hypothesis 3 was supported because the effect of burnout risk (β = −0.26, *p* < 0.01) on emotional engagement was significantly negative (small effect). Hypothesis 4 was supported because the effect of POS (β = 0.31, *p* < 0.01) on engagement was significantly positive (medium effect).

As regards indirect effects, the relationship between the moderator (Z) and the outcome (Y) must be known. Accordingly, the relationship between the moderator (lack of reciprocity) and the outcome (emotional engagement) was tested. Results showed a significant negative relationship between lack of reciprocity and emotional engagement (β = 0.19, *p* < 0.01, small effect). For the indirect effects, deductions were made based on the statistical significance of interaction terms shown in Mplus (Hernandez and Guarana, 2018). Results of the moderation analyses revealed that the interaction term (XZ), i.e., job demands × lack of reciprocity (β = 0.04, *p* = 0.72; 95% CI [−0.26, 0.18], CIs included zero) was not significant, accordingly, hypothesis 6a was not supported. Similarly, the interaction term POS × lack of reciprocity (β = 0.03, *p* = 0.68; 95% CI [−0.12, 0.18], CIs included zero) was not significant, and the moderation proposed in hypothesis 6b could not be supported. Furthermore, the interaction term burnout risk × lack of reciprocity (β = −0.12, *p* = 0.26; 95% CI [−0.32, 0.09], CIs included zero) was not significant, and the moderation proposed in hypothesis 6c could not be supported. Thus, the independent variables POS (safety and meaningfulness), burnout risk (availability), and lack of reciprocity (meaningfulness) as direct effects explained 39% of the variance in emotional engagement (*R*^2^ = 0.39).

For the portion of the model predicting psychological well-being (see Figure 2), the direct effects of burnout risk (β = −0.47, *p* < 0.01, medium effect) and emotional engagement (β = 0.27, *p* < 0.01, small effect) were respectively significantly negative and significantly positive. These relationships provided support for hypotheses 7 and 8. The two independent variables (burnout risk and emotional engagement) explained 42% of the variance in psychological well-being (*R*^2^ = 0.42).

Hypothesis 9 proposed that psychological well-being would be positively related to students’ reports on lecturers’ levels of performance. Due to low power and the lecturer performance scale’s demonstration of an insignificant regression path (close to 0) on well-being, the scale was removed from the path model.

The path model (using unstandardised path coefficients obtained from the Mplus analysis) tested three mediating effects. It is noted that where this study made mention of mediation or mediation analysis, this was done to test indirect effects. As proposed by hypothesis 10, it was tested if engagement mediated the relationship between burnout risk and psychological well-being. A negative and significant indirect effect of burnout risk on psychological well-being via engagement (β = −0.07; *p* = 0.03; 95% CI [−0.12, −0.01], CIs did not include zero) was found; therefore hypothesis 10 was supported. Further, hypothesis 11 was supported as the results revealed a negative and significant indirect effect of job demands on engagement via burnout risk (β = −0.12-; *p* = 0.01; 95% CI [−0.21, −0.03], CIs did not include zero). The path model included a *post hoc* hypothesis based on the work of Hakanen et al. (2006) and Russell et al. (2020) which was discussed earlier. This *post hoc* hypothesis (H12) proposed that burnout risk mediated the relationship between POS and emotional engagement. The proposal of hypothesis 12 was supported by the results which revealed a positive and significant indirect effect of POS on emotional engagement via burnout risk (β = 0.07; *p* = 0.02; 95% CI [0.01, 0.12], CIs did not include zero).

## Discussion

The purpose of this study was to answer the call of scholars for more studies on the topic of engagement (Bailey et al., 2017), and within the HE context, to explore the impact of work demands and resources on the engagement of academic staff (Najeemdeen et al., 2018) during the COVID-19 pandemic. The study thus set out to explore the interplay of conditions that stimulate the positive psychological construct of engagement (Kotera and Ting, 2019) by integrating Kahn’s (1990) theory on engagement with the JD-R model (Bakker and Demerouti, 2017) and other concepts such as reciprocity (Schaufeli et al., 1996) and POS (Eisenberger et al., 1986).

As regards engagement, Kahn’s (1990) theory focuses on understanding the objective properties of work contexts and roles and the importance of people’s experiences in these contexts. In explaining engagement, the JD-R model focuses on job characteristics (job resources and demands) and whether they involve psychological/physiological costs or are functional in achieving work goals (Bakker and Demerouti, 2017). The present study showed that the effects of burnout risk (availability) (hypothesis 3) and lack of reciprocity (meaningfulness) on emotional engagement were small, though significant. Findings pointed to a greater focus by academic staff on their experiences of the organisational context, in other words to POS as a job resource (hypothesis 4). This finding supported the finding of Schneider et al. (2018) that organisational practices had the strongest correlation with work engagement, even stronger than work attributes. The non-significant association between job demands and engagement (hypothesis 2) seems inconsistent with those who found a negative association between the constructs (e.g., Crawford et al., 2010; Han et al., 2020), however, previous studies (e.g., Gan and Gan, 2014) have found no significant association between job demands and engagement. Further, the present study’s finding may be regarded as corroborating the findings of May et al. (2004), Olivier and Rothmann (2007), Rothmann and Rothmann (2010), Rothmann and Welsh (2013) and Liu et al. (2021), who found that psychological meaningfulness was the strongest predictor of engagement. In this present study, POS involved aspects of both psychological meaningfulness and safety, which might explain the stronger association with engagement. Based on the findings of the study conducted by Liu et al. (2021) during the global COVID-19 pandemic, it might be that the academic staff participating in the present study perceived that organisational support functioned as an important safeguard against the negative impact of the pandemic on their experiences in the work context.

In the present study, consideration was given to lack of reciprocity (meaningfulness) being a moderator of the proposed antecedents to engagement, these include job demands, POS as job resources (safety and meaningfulness), and burnout risk (availability). However, none of these moderation interactions were found to be significant predictors of engagement (hypotheses 6a, b, and c). Reference may be made here to scholars’ (Andersson et al., 2014; Memon et al., 2019) proposed seven-step framework in conceptualising moderating relationships. Although most of the mentioned steps were considered, some, such as step number 6, to explore reverse interaction in which the independent variable might be the moderator should be ruled out theoretically, may have required more consideration. These findings indicate that the strength of lecturers’ perceptions of lack of reciprocity from student groups, did not weaken or otherwise strengthen the respective positive and negative influences of POS (safety and components of meaningfulness) and burnout risk (availability) on emotional engagement.

As regards burnout risk and well-being, findings from this study aligned with the review study of Halbesleben and Buckley (2004) in that job demands (time pressure; teaching vs research) seemed to be the main initiator of burnout risk (hypothesis 1 and 5). In explaining the stronger negative association between burnout risk and psychological well-being (hypothesis 7) when compared to the positive influence engagement had in improving psychological well-being (hypothesis 8), reference may be made to recent studies (e.g., Kadhum et al., 2020; Denning et al., 2021). These studies highlighted the negative effect of the COVID-19 pandemic, particularly on employees’ levels of burnout risk, and the ways in which the changes that the pandemic brought about threatened the psychological and overall well-being of people (Harju et al., 2021; Meyer et al., 2021).

The lecturer performance scale was removed from the structural model because the scale’s inclusion resulted in the model showing low power. The scale further demonstrated an insignificant regression path (close to 0) on well-being. Hypothesis 9 could thus not be examined. However, previous research suggested that the rating by students of teaching effectiveness might be misleading as students tend to evaluate educators based on popularity rather than on effectiveness (Obenchain et al., 2001); Tan et al. (2019) concurred, stating that students evaluate educators’ performance effectiveness based on their reactions to non-instructional or irrelevant characteristics, such as traits or attractiveness.

Lastly, findings from the study provided support for the mediation effects proposed. Hypothesis 10 showed complementary mediation (Zhao et al., 2010), in that the mediated effect (burnout risk × emotional engagement) and the direct effect were both significant and pointed in the same direction. These findings revealed that lecturers’ burnout risk had an indirect effect on their psychological well-being through the mediating role of emotional engagement. The findings suggest that experiencing high levels of burnout risk weakens the emotional engagement of academic staff, which in turn negatively influences their well-being. The *post hoc* hypothesis (hypothesis 12) also displayed complementary mediation (Zhao et al., 2010) in that the mediated effect (POS × burnout risk) and the direct effect were both significant and pointed in the same direction. The results suggest that the lecturers’ perception of organisational support and the indirect effects of burnout play an important role in shaping engagement. In both the above cases, the significant direct effects may suggest the existence of some omitted mediator which could be explored in future research (Zhao et al., 2010). Furthermore, results revealed that job demands had an indirect effect on the emotional engagement of academic staff through the mediating role of burnout risk (hypothesis 11). This offered a good example of indirect-only mediation and suggests that it is unlikely that there exist additional mediators (Zhao et al., 2010).

## Theoretical Contributions

This research study responded to the call for more studies to be conducted on the antecedents (Rothmann and Welsh, 2013) and topic of engagement, and to do so in alignment with the construct’s positive psychological roots (Bailey et al., 2017). The study aimed to make a contribution by exploring the conditions that enabled engagement specifically among academic staff within the HE context (Najeemdeen et al., 2018). For this purpose, this study also aimed to establish conceptual connections between some aspects of Kahn’s (1990) psychological conditions of meaningfulness, safety, and availability, and the JD-R model (Demerouti et al., 2001; Bakker and Demerouti, 2017), which is regarded as a well-being and job design framework (Rattrie et al., 2020). Furthermore, this study aimed to establish connections between Kahn’s (1990) theory on engagement and concepts relating to organisational support theory (Eisenberger et al., 1986) and perceptions of reciprocity (Schaufeli et al., 1996) to gain a better understanding of the conditions in an organisation that enable engagement and promote well-being in accordance with the tenets of positive psychology (Seligman et al., 2005).

## Implications of the Research

Although previous studies based on the JD-R model have highlighted five types of job resources (Xanthopoulou et al., 2007) (i.e., social support, autonomy, supervisory support, opportunities for professional development, and performance feedback) that are recognised as operating as antecedents to engagement in the majority of occupations, the present study demonstrated that, in alignment with organisational support theory, POS serves as a valued job resource (Kraimer and Wayne, 2004). POS not only taps into the five aforementioned categories (Eisenberger et al., 1997; Kurtessis et al., 2017) but further carries with it aspects that support psychological meaningfulness and safety, as conceptualised by Kahn (1990).

Through the integration of theory and literature on engagement (i.e., Kahn’s 1990 theory and the JD-R model), the study found that Kahn’s psychological conditions could be operationalised as POS (meaningfulness and safety), burnout risk (availability), and lack of reciprocity (meaningfulness). Findings indicated no relationship between job demands and emotional engagement but revealed a small negative and significant indirect effect of job demands on engagement via burnout risk. Furthermore, POS showed a stronger association with emotional engagement than did burnout risk and lack of reciprocity. With regard to this finding, reference can be made to Barrick et al.’s (2015) statement that strategic and deliberate management of organisational resources are required to foster an engaged workforce, as well as to Boikanyo and Heyns’s (2019) statement that organisations need to view engagement as a broad organisational strategy. Considering these findings, the practical implication of the present study’s findings is that it could assist university leaders in recognising the importance of creating conditions that enable the engagement of their staff. For example, universities could design policies and practices and consider strategies that are geared toward POS and that give employees the assurance that they are valued and regarded as important contributors to institutional objectives, particularly during times of change or crises.

Findings from the study also revealed that while burnout risk and emotional engagement explained 42% of the variance in psychological well-being, the negative effect of burnout risk was stronger than the positive effect of engagement. This finding highlights the importance that tertiary institutions (universities) should address burnout as it has implications for the psychological well-being of academic staff. University leaders could, therefore, consider strategies such as employee wellness/assistance programmes (online and face-to-face) to address psychosocial issues (e.g., burnout risk, work-/home-related stress). These programmes might not only provide the needed support to employees by addressing burnout risk but might also have the potential to create the positive perception among employees that their institution cares about their well-being and values them.

## Limitations and Suggestions for Further Research

Although the study had strengths (e.g., a solid theory-driven approach and the inclusion of reliable measures), it also had a few limitations. First, the use of manifest factor scores with error variance might have attenuated the path coefficients to some extent, although the effect should be small where measurement models show high reliability and factor determinacy coefficients, as was the case in this study. A second limitation of the study was its cross-sectional nature and its reliance on self-report data, making the study prone to common method variance (CMV) (Rindfleisch et al., 2008). Researchers have noted that method variance in organisational research accounts for less variance than previous studies have suggested (Lance et al., 2010). There has also been an ongoing debate regarding whether the presence and effects of CMV are of real concern for construct-valid self-report measures (Spector, 2006; Fuller et al., 2016). Nevertheless, future studies should implement efforts to mitigate variance. The present study implemented several strategies to mitigate some of the issues associated with cross-sectional data and CMV (Chang et al., 2010). For example, it followed the suggestions of Podsakoff et al. (2003) to use different scale formats and anchors for the different constructs that were measured in order to be in alignment with how the relevant measures had been developed (Podsakoff et al., 2003; Chang et al., 2010). These efforts were shown to reduce the likelihood of cognitive processing (Rindfleisch et al., 2008). Efforts were also made to ensure that the wording of questions was concise and clear by using more familiar concepts rather than concepts that could be perceived as complex or unfamiliar (Rodríguez-Ardura and Meseguer-Artola, 2020).

A third limitation was that data was collected over a period of 4 months to ensure a large enough sample size. Therefore, causal inferences cannot be made. Nevertheless, based on the tenets of the JD-R theory and the findings of previous longitudinal studies, it can be inferred that demands are predictive of burnout risk and that resources are predictive of engagement (see, for example, Hakanen et al., 2008; Sonnentag et al., 2010). Future studies may nonetheless consider a longitudinal design to gain a better understanding of the interplay and causal influences among the constructs. In addition, it may be worthwhile for future studies to consider these variables in a non-crisis state, such as when the COVID-19 pandemic has passed.

A fourth limitation was that the generalisability of the findings might be limited because all participants were academic staff from one South African tertiary institution. It is recommended that future studies explore these variables in different university settings locally or internationally and use a longitudinal design to validate causality among the variables. Lastly, as noted within the results section, power of 0.79 (*N* = 160; *df* = 10, effect = 0.10, α = 0.05) was obtained, thus statistical interaction effects lower than 0.10 were likely to not be recognised. With reference to the non-significant indirect and moderating effects, a limitation of the study may be that insufficient power existed to pick up lower-lying effect sizes. The notion is further supported by the relatively large confidence intervals reported for the statistically insignificant parameter estimates of the three moderator effects in the model. It is a common problem in studies reporting multiple moderating effects in the social sciences. Scholars noted that SEM models with multiple moderating effects require large samples to detect significant effects (Aguinis et al., 2017).

## Conclusion

Kahn (1990) noted the importance of understanding (and investigating) the degree to which people are psychologically present during moments or circumstances of performing a certain role, and what their emotional reactions are to both conscious and unconscious phenomena. The present research study attempted to apply Kahn’s (1990) theory on engagement by taking a closer look at the interplay of the psychological conditions (meaningfulness, safety, and availability) that stimulated the engagement of academic staff. The researcher provided support for Kahn’s theory on personal engagement by connecting Kahn’s psychological conditions with concepts focusing on the person-role relationship, such as those dealt with in the JD-R model, organisational support theory, and perceptions of reciprocity. The findings highlighted the importance of addressing these psychological conditions as they could lead to personal engagement. Further, the findings highlighted the implications of burnout risk and emotional engagement for the psychological well-being of academics.

It is hoped that the findings of this study might improve practices and policies within HE institutions and lead to a recognition of the importance for such practices and policies to be geared toward fostering engagement and well-being. Furthermore, the study’s findings might motivate university leaders to take note of the impact of POS and the need not only to lend the required support to academics in the face of dealing with various stressors but also to improve academics’ general engagement.

## Data Availability Statement

The original contributions presented in the study are included in the article/supplementary material, further inquiries can be directed to the corresponding author/s.

## Ethics Statement

This study was reviewed and approved by the Faculty of Economic and Management Sciences, University of Pretoria (Protocol number: EMS105/20). Obtaining participants’ informed consent formed part of the survey.

## Author Contributions

MR and CO conceived the presented idea. MR developed the theoretical linkages and wrote the manuscript. CO and PS supervised the project, provided critical feedback, and assisted in shaping the research. PS conducted the statistical analyses. All authors contributed to the final version of the manuscript.

## Conflict of Interest

The authors declare that the research was conducted in the absence of any commercial or financial relationships that could be construed as a potential conflict of interest.

## Publisher’s Note

All claims expressed in this article are solely those of the authors and do not necessarily represent those of their affiliated organizations, or those of the publisher, the editors and the reviewers. Any product that may be evaluated in this article, or claim that may be made by its manufacturer, is not guaranteed or endorsed by the publisher.
